# Pattern Recognition of Cognitive Load Using EEG and ECG Signals

**DOI:** 10.3390/s20185122

**Published:** 2020-09-08

**Authors:** Ronglong Xiong, Fanmeng Kong, Xuehong Yang, Guangyuan Liu, Wanhui Wen

**Affiliations:** 1 School of Electronic and Information Engineering, Southwest University, Chongqing 400715, China; xiongtaiyang@email.swu.edu.cn (R.X.); fanmengk@email.swu.edu.cn (F.K.); xuehong123@email.swu.edu.cn (X.Y.); liugy@swu.edu.cn (G.L.); 2 Chongqing Key Laboratory of Nonlinear Circuits and Intelligent Information Processing, Chongqing 400715, China

**Keywords:** e-learning, cognitive load, physiological measures, nervous response, pattern recognition

## Abstract

The matching of cognitive load and working memory is the key for effective learning, and cognitive effort in the learning process has nervous responses which can be quantified in various physiological parameters. Therefore, it is meaningful to explore automatic cognitive load pattern recognition by using physiological measures. Firstly, this work extracted 33 commonly used physiological features to quantify autonomic and central nervous activities. Secondly, we selected a critical feature subset for cognitive load recognition by sequential backward selection and particle swarm optimization algorithms. Finally, pattern recognition models of cognitive load conditions were constructed by a performance comparison of several classifiers. We grouped the samples in an open dataset to form two binary classification problems: (1) cognitive load state vs. baseline state; (2) cognitive load mismatching state vs. cognitive load matching state. The decision tree classifier obtained 96.3% accuracy for the cognitive load vs. baseline classification, and the support vector machine obtained 97.2% accuracy for the cognitive load mismatching vs. cognitive load matching classification. The cognitive load and baseline states are distinguishable in the level of active state of mind and three activity features of the autonomic nervous system. The cognitive load mismatching and matching states are distinguishable in the level of active state of mind and two activity features of the autonomic nervous system.

## 1. Introduction

The aim of effective instructional design is to help learners construct or automate knowledge schemas by means of specific strategies at certain learning circumstances [1,2,3]. For example, collaborative group study helps learners with task-specific knowledge get better learning outcomes than the novices [1]; incorporating positive emotional design principles into multimedia lessons helps learners perform better on a subsequent retention test [2]; instructional animations are superior to static graphics in short learning sections, but not in long learning sections [3]. The key for effective learning is the matching of cognitive load and working memory [4]. Otherwise, when cognitive load exceeds working memory capacity, cognitive overload leads to bad learning outcomes [4]. Therefore, it is meaningful to monitor cognitive load in the learning process. An experienced teacher can judge cognitive load through an observation of the learner’s behavior and learning outcome [3]. However, teachers are often absent in e-learning, urging people to explore automatic cognitive load detection methods by using physiological measures.

Previous researchers have found that skin conductance response, which is controlled by the sympathetic nervous system (SNS), is significantly associated with cognitive load condition [5,6]. Besides, heart rate variability (HRV), controlled by both branches of the autonomic nervous system (ANS), has shown sensitivity to cognitive task load, conditions of event rate and task duration [7]. In addition to autonomic nervous measures, central nervous indices, e.g., theta power and alpha suppression of the electroencephalography (EEG), are also valid objective measures of average cognitive load [8,9,10]. These findings support that cognitive effort in the learning process has nervous responses encoded in the variation of quantitative parameters of several physiological signals.

Many machine learning methods have been applied to cognitive load detection, as shown in Table 1 [11,12,13,14,15,16,17,18,19,20,21,22,23,24,25]. The main idea of these methods is as follows. Firstly, define two or three categories of cognitive load conditions. Secondly, extract a set of physiological parameters as the features of cognitive load. Thirdly, train certain classifiers with data samples acquired from groups of subjects. Finally, obtain computational models for the pattern recognition of cognitive load through performance comparison of the classifiers. The strengths of the computational models are usually determined by the amount of data samples and subjects, the dimension of feature sets, the accuracy of the classifiers, and the validation methods of the models. For a given data sample set, a lower dimension of the feature set and a higher accuracy of the classifier and subject-independent validation method lead to a better pattern recognition model. As shown in Table 1, the subject-independent accuracy of the cognitive load recognition models still needs to be improved by means of more effective features, classifiers, or physiological signals.

In order to accurately recognize cognitive load conditions, the current work explored a large set of initial features of HRV and EEG, and selected a low-dimension critical feature subset by using sequential backward selection (SBS) and particle swarm optimization (PSO) algorithms. We trained several common classifiers with selected feature subset, and finally constructed effective pattern recognition models of cognitive load through subject-independent validation. Although previous work has shown that no explicit HRV parameters were continuously correlated with EEG parameters [26], we hypothesize that HRV and EEG parameters are complementary with each other, and their combination can improve the recognition of cognitive load conditions.

## 2. Materials and Methods

The dataset used in this work is available on Physiobank, and has been contributed by Igor Zyma, Sergii Tukaev, and Ivan Seleznov [27,28]. Firstly, necessary information about this dataset, e.g., subjects and mental task procedure, is introduced in this section. Secondly, we regrouped the data to form two binary classification problems of cognitive load. Thirdly, HRV and EEG parameters commonly used in literature were extracted as physiological features of cognitive load conditions. Lastly, we introduced the methods of feature selection and the criteria of classification accuracy.

### 2.1. Subjects

In total, 66 healthy right-handed volunteers (47 women and 19 men) were initially involved in the study. All subjects were 1st–3rd year students of the Taras Shevchenko National University of Kyiv, aged 18 to 26 years (18.6 ± 0.87 years). The subjects were eligible to enroll in the study if they had normal or corrected-to-normal visual acuity, normal color vision, and had no clinical manifestations of mental or cognitive impairment or verbal or non-verbal learning disabilities. Exclusion criteria were the use of psychoactive medication, drug or, alcohol addiction, and psychiatric or neurological complaints.

### 2.2. Experiment Procedure

The mental arithmetic task for inducing cognitive load was to continuously subtract the two-digit number from the four-digit number, and the accurate calculation times of each subject in four minutes were counted out after the arithmetic task. The subjects were rated according to the number of accurate calculations, and they were divided into two groups [27,28]. Group “G” had 24 subjects who performed good quality count (number of accurate calculations in 4 min: 21 ± 7.4); Group “B” had 12 subjects who performed bad quality count (number of accurate calculations in 4 min: 7 ± 3.6) [27,28]. Before the 4-min mental arithmetic task began, there were 3 min of baseline state recorded from each subject. ECG and EEG data were acquired throughout the experiment, but only the 3-min baseline data and the data in the first-minute calculation were kept in the dataset [27,28].

The EEGs were recorded monopolarly using the Neurocom EEG 23-channel system. The silver/silver chloride electrodes were placed on the scalp according to the International 10/20 scheme. All electrodes were referenced to the interconnected ear reference electrodes. Noise and artifacts in EEG data were removed by a 0.5 Hz cut-off frequency high-pass filter, a 45 Hz low-pass filter, a 50 Hz power-line notch filter and an independent component analysis (ICA) method [27,28].

**Figure 1:**
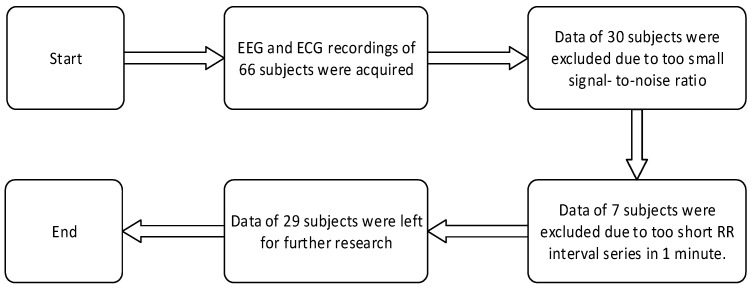
The flowchart of the data selection and exclusion.

We applied an adaptive threshold method based on wavelet decomposition to locate the position of R peaks in the ECG data and to obtain the RR interval series [29]. Due to the variation of heartbeat speed of different subjects, the number of RR intervals in one minute varies from 60 to 120. In order to obtain a compromise between the length of RR interval series and the number of subjects included in the dataset, we set 80 as the length of the RR interval series, and those without 80 RR intervals in one minute were excluded from the original dataset, keeping the data of 29 subjects (21 women and 8 men) for further analysis. The data selection and exclusion process is shown in Figure 1.

### 2.3. Grouping Rules

We aimed to solve two binary classification problems: cognitive load (CL) state vs. baseline (BL) state and cognitive load mismatching (CLMM) state vs. cognitive load matching (CLM) state. These two binary classification problems have practical values in e-learning. Distinguishing CL from BL helps the e-learning system to tell whether the learner is currently learning. Recognition of CLMM and CLM also helps the e-learning system to judge whether the learner is effectively learning. For the CL vs. BL classification problem, we divided the data into CL and BL groups: the CL group corresponding to data of the first-minute calculation, and the BL group corresponding to data of one-minute baseline. For the CLMM vs. CLM classification problem, we divided the data into CLMM and CLM groups: the CLMM group having first-minute calculation data of the subjects with bad quality count, and the CLM group having first-minute calculation data of the subjects with good quality count. We took effort to construct three different pattern recognition models for each of the above two binary classification problems, and these models were based on different options of physiological signals. The detailed information of the models is shown in Table 2.

### 2.4. Feature Extraction

In order to quantify the ANS activity, we extracted 27 linear and nonlinear parameters from the RR interval series as features of cognitive load conditions. These parameters were commonly applied to HRV analysis in literature [30,31,32,33]. Besides, six commonly used EEG parameters which measured the central nervous system (CNS) activity, e.g., powers of sub-band brainwaves [34], were also applied as features of cognitive load conditions. The HRV and EEG features are respectively shown in Table 3 and Table 4.

### 2.5. Balanced Sample Sets

As shown in Table 2, the sample sets of CLMM and CLM are unbalanced, which may cause the bias of the pattern recognition model to the majority samples. In order to avoid such bias, we adopted the Borderline-SMOTE1 algorithm to oversample the minority samples [35], so that the sample size of the CLMM group expanded to 18, the same to that of the CLM group.

### 2.6. Feature Selection and Classification

The original 27 HRV parameters and 6 EEG parameters were commonly applied to the analysis of ANS and CNS activities in literature [30,31,32,33,34]. However, they are not specific to the pattern recognition of cognitive load conditions. In terms of accuracy and computational efficiency, some features can even be a burden of the classification problems. Therefore, feature selection is necessary to find out the feature subset that is critical to the classification problems. The feature selection process is a combinatorial optimization problem to find a vector ${\vec{x}}^{*}$ in the feature space *X*, so that

(1) $$\forall \vec{x}\in X,\begin{array}{cc} & f( \end{array}{\vec{x}}^{*})=\min f\left(\vec{x}\right)$$

where $\vec{x}={\left[x_{j}\right]}_{1\times M}$ is a vector consisting of *M* features, with $x_{j}=1$ denoting the *j*th feature is selected, and $x_{j}=0$ denoting the *j*th feature is not selected. f(⋅) is the evaluation function of the selected feature subset. In order to keep the complementary information among the features in the subset, we chose sequential backward selection (SBS) algorithm and particle swarm optimization (PSO) algorithm to perform feature selection [36,37]. SBS eliminates the least important feature from the subset at each iteration, until there is only one feature left in the subset. On the one hand, SBS is good at reducing the dimension of the feature subset. On the other hand, PSO has a strong ability of global optimization [38]. Therefore, when combining PSO and SBS, we firstly applied PSO to get an optimal feature subset which has the best evaluation function value in the searching process, and then used SBS to further reduce the dimension of the optimal feature subset. The quality of selected feature subset in each iteration of SBS and PSO process was evaluated by evaluation functions, i.e., *f*_1_ for SBS and *f*_2_ for PSO, which are calculated as in Equations (2) and (3):

(2) $$f_{1}=\frac{\sum_{n=1}^{N}n_{\mathrm{correct}}}{n_{\mathrm{total}}}$$

(3) $$f_{2}=\alpha \times \frac{\#Features}{\#All\;Features}+\left(1-\alpha \right)\times \frac{ErrorRate}{ER}$$

where *N* is the number of subjects (*N* = 36 for CLMM vs. CLM, and *N =* 29 for CL vs. BL), $n_{\mathrm{correct}}$ is the number of correctly classified samples of one subject, and $n_{\mathrm{total}}$ is the number of samples of all subjects. α is the weighting coefficient, which is empirically set as α = 0.8. Moreover, *#Features* means the number of features currently selected, and *#All Features* is the total number of initial features. *ErrorRate* and *ER* respectively represent the mean error rates obtained by currently selected features and all features. $n_{\mathrm{correct}}$, $n_{\mathrm{total}}$, *ErrorRate* and *ER* were calculated in leave-one-subject-out cross validation. The leave-one-subject-out cross validation is to leave the samples belonging to one subject as the test set, and to repeat such test until the samples of every subject are tested. In the PSO feature selection process, the population size *P =* 30, the maximum iteration *T* = 50 in Model A and D and *T* = 100 in other models, the inertia weight *ω* = 0.7298, and the acceleration constants $C_{1}=\;C_{2}=2$.

**Table 4:** EEG Features of Cognitive Load.

EEG Index	Description	Relation with CNS Activity
DP	Delta band (1–4 Hz) power	A measure of unconscious mind [34].
TP	Theta band (4.1–5.8 Hz) power	A measure of subconscious mind [34].
AP	Alpha band (5.9–7.4 Hz) power	A measure of relaxed mental state [34].
BP	Beta band (13–19.9 Hz) power	A measure of active state of mind [34].
GP	Gamma band (20–25 Hz) power	A measure of hyper brain activity [34].
WE	Wavelet entropy	A measure of energy distribution of EEG at different scales [39].

In this work, we chose DB4 as wavelet packet decomposition function with the scale of 7 to calculate the WE.

Four kinds of classifiers, namely support vector machine (SVM, kernel function types: ‘quadratic’ and ‘rbf’), K-nearest neighbor (KNN) and decision tree (DT), were adopted to solve the above-mentioned binary classification problems, and they were trained by the samples of N−1 subjects. Sensitivity (Sens.), specificity (Spec.), precision (Prec.), accuracy (Acc.), F1-score (F1) and the area under receiver operating characteristic (AUC) of the leave-one-subject-out cross validation were calculated to evaluate the performance of each classifier, as shown in Equations (4)–(9):

(4) $$\mathrm{Sens}=\frac{TP}{TP+FN}$$

(5) $$\mathrm{Spec}=\frac{TN}{TN+FP}$$

(6) $$\mathrm{Prec}=\frac{TP}{TP+FP}$$

(7) $$\mathrm{Acc}=\frac{TP+TN}{TP+FP+TN+FN}$$

(8) $$F1=\frac{2*Sens*Spec}{\left(Sens+Spec\right)}$$

(9) $$\mathrm{AUC}=\frac{1}{2}\left(\frac{TP}{TP+FN}+\frac{TN}{TN+FP}\right)$$

For each of the binary classification problems, we set samples in the CL and CLM categories as the positive ones, and samples in the BL and CLMM categories as the negative ones. In Equations (4)–(9), *TP* denotes the number of correctly classified samples in the positive category, *TN* denotes the number of correctly classified samples in the negative category, *FP* is the number of incorrectly classified samples in the negative category, and *FN* is the number of incorrectly classified samples in the positive category.

## 3. Results

### 3.1. Parameter Settings of Entropy Features

The value of entropy index depends on the settings of embedding dimension *m*, tolerance threshold *r* and delay time τ. The delay time τ was calculated with the mutual information method proposed in literature [40]. For the embedding dimension *m* and tolerance threshold *r*, we firstly set an initial variation range of *m* and *r*, and then determined the values of *m* and *r* through Mann–Whitney U test. The appropriate values of *m* and *r* resulted in the entropy indices, which were significantly different between the positive group (CL or CLM) and the negative group (BL or CLMM). The detailed parameter settings are shown in Table 5.

### 3.2. Results of Feature Selection

**Figure 2:**
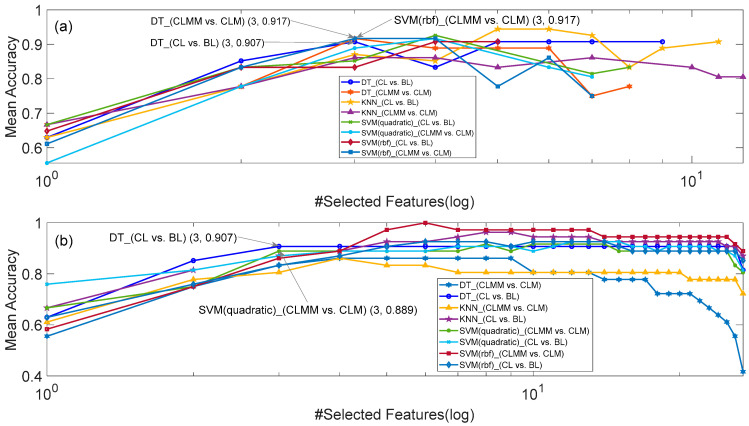
The results of HRV feature selection for Models A and D. (**a**) SBS and PSO, and (**b**) SBS.

**Figure 3:**
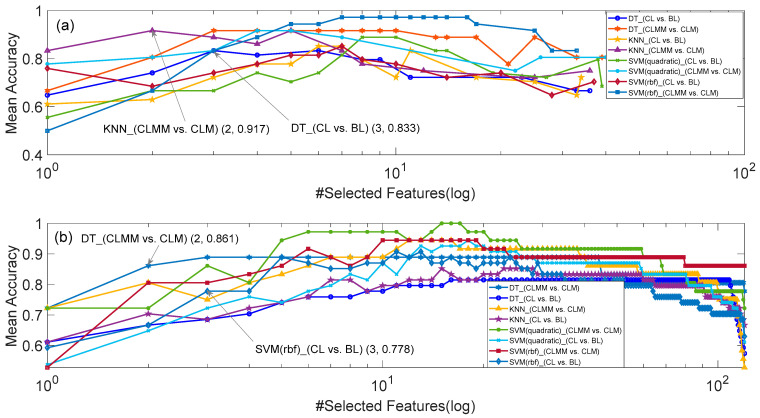
The results of EEG feature selection for Models B and E. (**a**) SBS and PSO, and (**b**) SBS.

**Figure 4:**
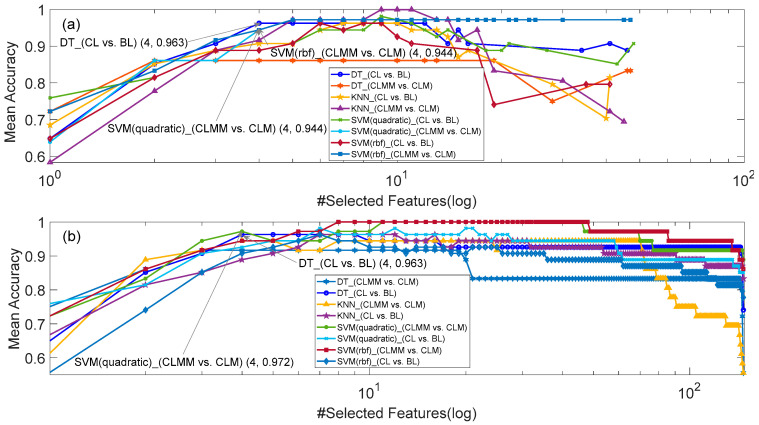
The results of EEG+HRV feature selection for Models C and F. (**a**) SBS and PSO, and (**b**) SBS.

Figure 2, Figure 3 and Figure 4 respectively show the number of selected features and corresponding mean accuracy in the process of feature selection. Although the evaluation function of PSO in Equation (3) takes feature dimension into consideration, the dimension of the best feature subset found by PSO is still high, e.g., 6 for KNN (CL vs. BL) in Figure 2a, 7 for SVM (rbf) (CLMM vs. CLM) in Figure 3a, and 9 for KNN (CLMM vs. CLM) in Figure 4a. Considering the relatively small sample amount in the dataset, the higher dimension of feature set leads to higher risk of over fitting.

Therefore, we made a compromise between the accuracy of the classifier and the dimension of selected features, i.e., decreasing the dimension of selected features at the cost of a small decrease of mean accuracy. As marked in Figure 2, Figure 3 and Figure 4, if we constrain the dimension of the feature subset to be no more than 4, the highest mean accuracies with HRV features, EEG features and HRV+EEG features, were respectively obtained by DT (CL vs. BL and CLMM vs. CLM) and SVM (rbf) (CLMM vs. CLM) in Figure 2, KNN (CLMM vs. CLM) and DT (CL vs. BL) in Figure 3, and SVM_quadratic (CLMM vs. CLM) and DT (CL vs. BL) in Figure 4. Compared with the SBS algorithm alone, PSO and SBS found better 3-dimension feature subsets for models B, D and E. As shown in Table 6, the cost of doing this is that the computation time increases more than ten times.

The performance indices of the classifiers in Models A–F are shown in Table 6. Compared with single-modal signal (HRV or EEG), the fusion of HRV and EEG obtained better pattern recognition performances for CL vs. BL and CLMM vs. CLM problems. In Models C and F, we can see that the features of EEG and those of HRV are complementary to each other, so that the combination of EEG and HRV features improves the distinguishability of the positive and negative samples, verifying the hypothesis made in the introduction section. The confusion matrices of Models C and F are shown in Table 7. The bold parts in Table 6 and Table 7 represent the best classification results for each model.

### 3.3. Validation with E-Learning Data

We acquired e-learning data from real-world math courses for model validation. Five subjects took our e-learning math course and had their EEG and ECG data recorded. As our EEG device had 128 channels, the sub-band brainwave energy was very different from that of the 23-channel EEG data in literature [27,28]. Therefore, we only used the ECG data to validate our Model A. Each subject provided 10 min of ECG data, including 5 min of baseline state and 5 min of e-learning state. After eliminating noisy data, the sample size of the validation data set is 41 (21 of CL state and 20 of BL state). Each sample was described as 3-dimension vector of the critical ECG features of Model A, i.e., Area, LF and ApEn. Finally, we got 65.5% F1 score of the validation accuracy of Model A in the real e-learning status, and the confusion matrix is shown in Table 8.

## 4. Discussion

Compared with the previous research in Table 1, our work not only considered the CL vs. BL classification problem, but also explored the pattern recognition of CLMM vs. CLM. For the CL vs. BL classification problem, the best subject-independent results of the previous research are those in literature [11], i.e., 90% of Acc, 86% of Sens. and 95% of Spec. with SD1, SD2 and a complex measure of HRV named En(0.2). The best subject-independent results of CL vs. BL classification in this work are given by the DT classifier of Model C in Table 6. As shown in Table 6, better performance indices than those in [11] were obtained, with three HRV features and one EEG feature. If we use the same dimension of features to that in [11], the SVM (quadratic) classifier with three features, namely AP_O1, GP_O1 and Mean, got the Acc., Sens. and Spec. indices of 90.7%, 96.3% and 85.2%, respectively. For the CLMM vs. CLM problem, we use two HRV features and two EEG features to get better classification results than those in literature [19]. By using the real e-learning ECG data, we got a validation accuracy of 65.5% F1 score, much higher than that of a random guess.

It is worth noting that the relatively small amount of samples and subjects has limited further exploration of the generalization performance of the models proposed in this work. Although the validation accuracy of Model A is better than that of random guess, it is far away from the requirement of real application. There are two ways to improve the accuracy of CL vs. BL and CLMM vs. CLM classification. The first one is to enlarge the amount of data samples, and the second one is to use other math tasks which elicit CLMM and CLM states of the college students (i.e., the subjects). In our validation data acquisition, we found that subtracting a two-digit number from a four-digit number was too easy for the college students, and the CLMM state failed to be elicited.

## 5. Conclusions

By using the combination of one EEG feature (BP_F4) and three HRV features (Mean, LF and ApEn), the DT classifier has classified the CL and the BL states with the accuracy of 96.3%, showing that the CL and BL states are distinguishable in the level of active state of mind, the average level of ANS activity, the combined activities of SNS and PNS, and the competition between SNS and PNS. For the classification of CLMM and CLM states, the SVM (quadratic) classifier, two EEG features (BP_T4, BP_O1) and two HRV features (MFD, TFC) have obtained the accuracy of 97.2%, showing that the CLMM and CLM states are distinguishable in the level of active state of mind, the total and the average fluctuation of ANS activity. The current work has application value in practice, because it provides an objective quantitative method for the monitoring of cognitive load conditions in e-learning.

## Figures and Tables

**Table 1:** Related work in cognitive load recognition.

Study	# Subjects	# Features	# Categories	Classifier	Signals	Best Accuracy	Validation Approach
Hasanbasic [11]	10	12	3	SVM	ECG, EDA	91.00%	SD
Melillo [12]	42	3	2	LDA	ECG	90.00%	SI
Cheema [13]	30	5	2	LS-SVM	PCG	96.67%	SD
Wang [14]	10	32	2	PCA, SVM	EEG	97.14%	SD
Al-Shargie [15]	22	9	2	SVM	EEG, fNIRS	95.10%	SD
McDuff [16]	10	7	2	Naïve Bayes	PPG (HR, HRV, BR)	86.00%	SI
Ahn [17]	14	4	2	SVM	ECG, EEG	87.50%	SD
Xia [18]	22	4	2	PCA, SVM	EEG, ECG	79.54%	SD
Dimitrakopoulos [19]	28	23	2	SVM	EEG	86.00%	SD
Yu [20]	20	4	2	ELM	ECG	84.75%	SI
Wang [21]	160	-	2	LFDM, XGBoost	ECG, PPG	97.2%	-
Das Chakladar [22]	48	6	2	BLSTM-LSTM	EEG	86.33%	-
Barua [23]	66	42	2	Random Forest	HRV, GSR, RESP	78.00%	SD
Plechawska [24]	11	52	3	KNN	EEG	91.50%	SI
Fan [25]	20	5	3	SVM, PCA	EEG, ECG	80.00%	SI

SD: subject-dependent. In this case, samples belonging to one subject appear both in the training dataset and in the validation dataset, usually causing over-optimistic accuracy of the classifier. SI: subject-independent, which means subjects and samples belonging to the validation dataset are totally new to the trained classifier. LS-SVM: least-square support vector machine; PCG: phonocardiography; PPG: photoplethysmography; ELM: extreme learning machine; LFDM: linear feature dependency modeling; XGBoost: eXtreme Gradient Boosting; BLSTM-LSTM: a combination of bidirectional long short-term memory (BLSTM) and long short-term memory (LSTM) networks. PCG: phonocardiography; PPG: photoplethysmography; EDA: electrodermal activity; fNIRS: functional near-infrared spectroscopy; HR: heart rate; BR: breathing rate; RESP: respiration. #: number.

**Table 2:** Grouping rules.

Group	# Subjects	# Male	# Female	Model Name	Physiological Signal
CL vs. BL	27 vs. 27	8 vs. 7	19 vs. 20	Model A	HRV
				Model B	EEG
				Model C	HRV and EEG
CLMM vs. CLM	9 vs. 18	3 vs. 5	6 vs. 13	Model D	HRV
				Model E	EEG
				Model F	HRV and EEG

Among the data of 29 subjects, the BL data of two subjects and the CL data of another two subjects were heavily distorted by noise and eliminated from the dataset. #: number.

**Table 3:** HRV features of cognitive load.

Indices	Description	Relation with ANS Activity
SDRR	Standard deviation of RR intervals	A measure of HRV in time domain [26], which reflects the activities of SNS and PNS, mainly SNS activity [27].
RMSSD	Square root of the mean squared differences of successive RR intervals	A measure of HRV at one adjacent beat scale, which reflects the vagal activity [27].
Mean	Mean of RR intervals	A measure of the average level of ANS activity [26].
Area	Summation of RR intervals	A measure of total amount of ANS activity in time domain.
MFD	Mean of the first differences of RR intervals	A measure of HRV at one adjacent beat scale, which reflects the average fluctuation of ANS activity [25].
pNN20	Proportion of differences between successive RR intervals longer than 20 ms	A measure of HRV in time domain, which reflects the fluctuation of ANS activity.
pNN10	Proportion of differences between successive RR intervals longer than 10 ms	A measure of HRV in time domain, which reflects the fluctuation of ANS activity.
HRVC	Heart rate variation coefficient, calculated by the ratio of SD to Mean	A measure of normalized fluctuation of ANS activity.
VLF	The power of RR intervals between 0 Hz and 0.04 Hz	A measure of SNS activity [28].
LF	The power of RR intervals between 0.04 Hz and 0.15 Hz	A measure of combined activities of SNS and PNS [26,27].
HF	The power of RR intervals between 0.15 Hz and 0.4 Hz	A measure of PNS activity [26,27].
TOTPWR	The power of RR intervals between 0 Hz and 0.4 Hz	A measure of total amount of ANS activity in frequency domain [26].
HF/(LF+HF)	The ratio of HF/(LF+HF)	A measure of normalized PNS activity.
LF/(LF+HF)	The ratio of LF/(LF+HF)	A measure of normalized PNS+SNS activity [26].
LF/HF	The ratio of LF/HF	A measure of the balance between SNS and ANS [27].
Entropy	PeEn, ApEn, MFEn, SampEn	Measures of the complexity of RR interval series caused by competition between SNS and PNS [27].
DFA (α1, α2, α1/α2)	Detrend fluctuation analysis	Measures of the fractal properties of RR interval series caused by competition between SNS and PNS [27].
TFC	Total fluctuation coefficient	A measure of the fluctuation of ANS activity in scales 1~*M* [33]. We set *M* = 10 in the current work.
PP (SD1, SD2, SD1/SD2)	Poincaré Plot	Measures of short-term and long-term HRV, which reflects the fluctuation of ANS activity [26,27].
RLHE	Range of the local Hurst exponents	A measure of the complexity of RR interval series, which is controlled by competition between SNS and PNS [25].

PeEn: permutation entropy; ApEn: approximate entropy; SampEn: sample entropy; MFEn: multiscale fuzzy measure entropy; PNS: parasympathetic nervous system; α1 and α2 were calculated with the small scale (4 ≤ n ≤ 16) and large scale (16 ≤ n ≤ 32), respectively.

**Table 5:** Settings of entropy parameters based on MANN–WHITNEY U test.

Feature	Group	Mean ± SD	Embedding Dimension	Tolerance Threshold	Sig.	Description
ApEn	CL	0.67 ± 0.16	*m* = 2	*r* = 0.4 × SDRR	0.002	*m* varies from 1 to 3, and *r* varies from 0.1 × SDRR to 0.9 × SDRR
	BL	0.78 ± 0.14				
	CLMM	0.62 ± 0.12	*m* = 2	*r* = 0.6 × SDRR	0.041	
	CLM	0.47 ± 0.18				
SampEn	CL	1.17 ± 0.33	*m =* 1	*r* = 0.3	0.01	*m* varies from 1 to 3, and *r* varies from 0.1 to 0.9
	BL	1.39 ± 0.29				
	CLMM	0.70 ± 0.16	*m* = 2	*r* = 0.6	0.03	
	CLM	0.52 ± 0.21				
PeEn	CL	0.59 ± 0.03	*m =* 6	-	0.009	*m* varies from 3 to 7, and *τ* is calculated by mutual information method
	BL	0.61 ± 0.02				
	CLMM	0.97 ± 0.13	*m* = 3	-	0.017	
	CLM	0.93 ± 0.40				
MFEn	CL	0.33 ± 0.18	*m* = 1	*r* = 0.1	<0.001	*m* varies from 1 to 3, and *r* varies from 0.1 to 0.9. The scale of CL vs. BL and CLMM vs. CLM are 5 and 2, respectively
	BL	0.50 ± 0.13				
	CLMM	1.25 ± 0.16	*m* = 3	*r* = 0.2	0.015	
	CLM	1.02 ± 0.27				

Sig.: significance of Mann–Whitney U test.

**Table 6:** Critical feature subsets of models A–F and performance indices of the classifiers.

Model	Classifier	Critical Feature Subset	M_fs_	T_fs_ (min)	F1	Prec. (%)	Sens. (%)	Spec. (%)	AUC	Acc. (%)
Model A	SVM_q	Area, LF, HF/(LF+HF)	SBS	11.9	0.87	83.3	92.6	81.5	0.87	87.0
	SVM_r	RMSSD, LF, MFEn	SBS	10.4	0.83	82.1	85.2	81.5	0.83	83.3
	KNN	Area, LF, LF/HF	SBS	9.6	0.86	81.3	96.3	77.8	0.87	87.0
	**DT**	**Area, LF, ApEn**	**SBS**	**10.6**	**0.91**	**92.3**	**88.9**	**92.6**	**0.91**	**90.7**
Model B	SVM_q	AP_Pz, BP_F7, BP_O2	SBS	29.3	0.72	71.4	74.1	70.4	0.72	72.2
	SVM_r	DP_F8, AP_Fp1, BP_Pz	SBS	25.8	0.78	75.9	81.5	74.1	0.78	77.8
	KNN	DP_T3, TP_F8, AP_O1	PSO and SBS	1455.3	0.72	73.1	70.4	74.1	0.72	72.2
	**DT**	**AP_Fp2, AP_Pz, BP_O1**	**PSO and SBS**	**2234.7**	**0.82**	**78.1**	**92.6**	**74.1**	**0.83**	**83.3**
Model C	SVM_q	AP_O1, AP_A2A1, GP_O1, Mean	SBS	167.8	0.93	92.6	92.6	92.6	0.93	92.6
	SVM_r	WE_P3, Area, LF, ApEn	SBS	198.5	0.91	92.3	88.9	92.6	0.91	90.7
	KNN	TP_O1, Mean, LF, ApEn	PSO and SBS	762.9	0.90	86.7	96.3	85.2	0.91	90.7
	**DT**	**BP_F4, Mean, LF, ApEn**	**SBS**	**178.5**	**0.96**	**93.1**	**100**	**92.6**	**0.96**	**96.3**
Model D	SVM_q	MFD, SampEn, MFEn	SBS	7.9	0.88	100	77.8	100	0.89	88.9
	**SVM_r**	**CVrr, SD1, SD1/SD2**	**PSO and SBS**	**515.8**	**0.91**	**100**	**83.3**	**100**	**0.92**	**91.7**
	KNN	ApEn, SD1, SD1/SD2	PSO and SBS	558.3	0.85	93.3	77.8	94.4	0.86	86.1
	**DT**	**HF/(LF+HF), α2/α1, TFC**	**PSO and SBS**	**555.0**	**0.92**	**94.1**	**88.9**	**94.4**	**0.92**	**91.7**
Model E	SVM_q	DP_T4, AP_Pz,	PSO and SBS	1207.0	0.80	86.7	72.2	88.9	0.81	80.6
	SVM_r	WE_F4, WE_F7	SBS	29.0	0.78	73.9	94.4	66.7	0.81	80.6
	**KNN**	**DP_Cz, BP_F3**	**PSO and SBS**	**1141.8**	**0.92**	**94.1**	**88.9**	**94.4**	**0.92**	**91.7**
	DT	DP_T6, GP_T4	SBS	35.2	0.85	81.0	94.4	77.8	0.86	86.1
Model F	**SVM_q**	**BP_T4, BP_O1, MFD, TFC**	**SBS**	**114.0**	**0.97**	**100**	**94.4**	**100**	**0.97**	**97.2**
	SVM_r	GP_Fz, MFD, SampEn, SD2	SBS	125.8	0.94	94.4	94.4	94.4	0.94	94.4
	KNN	GP_T4, MFD, PeEn, TFC	SBS	200.8	0.94	100	88.9	100	0.94	94.4
	DT	AP_T4, LF, TFC, SD1/SD2	SBS	149.3	0.92	89.5	94.4	88.9	0.92	91.7

SVM_q: quadratic polynomial as kernel function of SVM. KNN: k-nearest neighbors using Euclidean distance weighting. SVM_rbf: rbf as kernel function of SVM. Fp2, F7, Pz, T3, C3, A2, A1, F4, T4, T5, F8, C4, O1, O2, F3, Fp1, Cz, P4 and T6 represent various channels of EEG signal. Naming rules for EEG features: feature name _ channel name. M_fs_: Method of feature selection. T_fs_: Time of feature selection.

**Table 7:** The confusion matrices of Models C and F.

Model	Classifier	M_fs_	Classified as	CL	BL	CLMM	CLM
Model C	SVM_q	SBS	CL	92.6%	7.4%	-	-
			BL	7.4%	92.6%	-	-
	SVM_r	SBS	CL	88.9%	11.1%	-	-
			BL	7.4%	92.6%	-	-
	KNN	SBS and PSO	CL	96.3%	3.7%	-	-
			BL	14.8%	85.2%	-	-
	**DT**	**SBS**	**CL**	**100**	**0%**	-	-
			**BL**	**7.4%**	**92.6%**	-	-
Model F	**SVM_q**	**SBS**	**CLMM**	-	-	**100%**	**0%**
			**CLM**	-	-	**5.6%**	**94.4%**
	SVM_r	SBS	CLMM	-	-	94.4%	5.6%
			CLM	-	-	5.6%	94.4%
	KNN	SBS	CLMM	-	-	100%	0%
			CLM	-	-	11.1%	88.9%
	DT	SBS	CLMM	-	-	88.9%	11.1%
			CLM	-	-	5.6%	94.4%

**Table 8:** The confusion matrix of validation using e-learning data.

Classifier	Classified as	CL	BL
DT	CL	55.0%	45.0%
	BL	19.0%	81.0%
